# Applications of an Equity Framework in COVID-19 Vaccine Trial and Distribution Planning

**DOI:** 10.1089/heq.2021.0122

**Published:** 2022-01-25

**Authors:** Alysia Davis, Dara D. Mendez

**Affiliations:** ^1^ Equity Consultant and Maternal Child Health Advocate, Best4Baby, LLC, Pittsburgh, Pennsylvania, USA.; ^2^ Department of Epidemiology, Graduate School of Public Health, University of Pittsburgh, Pittsburgh, Pennsylvania, USA.

**Keywords:** COVID-19, racism, systemic oppression, vaccine research, vaccine trial, trustworthiness

## Abstract

There are a disproportionate number of cases, hospitalizations, and deaths among Black and Latinx communities, a result of a history of structural racism and exploitation. An equity framework and approach are critical but have been lacking in the COVID-19 response, including vaccine dissemination. We provide an overview and application of remove, repair, remediate, restructure, and provide (R4P), an equity framework, in examining COVID-19 vaccine trial development and related interventions. R4P is an equity framework and tool that applies critical race theory, intersectional, and dimensionality in planning, assessment, and research for creating action to eliminate inequities.

There have been 39 million cases and >600,000 deaths from COVID-19 in the United States since the end of August 2021.^1^ The nation is currently experiencing a surge in cases due to low vaccination rates and the deadly and highly contagious delta variant. Black populations are two times as likely to contract COVID-19, five times as likely to be hospitalized, and two times as likely to die.^2^ The first month of COVID-19 vaccine distribution in the United States, of the 12 million people vaccinated, only 5.4% identified as Black and 11.5% as Hispanic, whereas White American's made up 60.4% of those vaccinated.^3^ President Joe Biden announced all Americans would be eligible to receive the COVID-19 vaccine by May 2021. While access for the COVID-19 vaccine has increased due to federal policies, the vaccination rates for Black and Hispanic people remain low.^4^

A recent report found that Black adults expressed less trust in medical providers, the medical system, and were less likely to participate in COVID-19 clinical trials and being vaccinated.^5^ The distrust is a result of centuries of documented exploitation by medical and research institutions and by systemic exclusion from clinical research.^5^ To address the lack of diverse representation in clinical research and to ensure the COVID-19 vaccine's success across populations, the medical and health professional communities must work to build trust with Black and Latinx communities and eliminate exploitative and inequitable practices.

In cocreating new processes, it is essential to apply and integrate an equity framework throughout all phases of clinical trial planning, and vaccine distribution. Members of the vaccine research and implementation team should include Black, Latinx, and multilingual individuals, at all levels of the study and distribution process. Establishing coalitions creates an accountability body to provide oversight and transparency but as coleaders in cocreating the plans and processes throughout the duration of trials and vaccine dissemination.

The R4P (remove, repair, remediate, restructure, and provide) is an equity framework and tool that applies critical race theory, intersectional, and dimensionality in planning, assessment, and research for creating action to eliminate inequities.^6^ The tool was designed based on the historical and contemporary experiences and contexts of Black Americans, although can be adapted and applied in considering multiple populations.^6^ The R4P framework can be applied with COVID-19 clinical trials, vaccine dissemination, and other teams involved in the pandemic response.

The tenets of the framework allow for engagement in multiple domains at the same time. We present the framework along with guiding questions and action steps teams should consider when approaching this work. This framework is applicable to COVID-19 vaccine development and distribution but is valuable for enhancing our approach to public health and equity.

**REMOVE** entails eliminating the *structures, attitudes, and practices that disproportionately negatively impact specific groups with respect to race, gender, socioeconomic status (or other social groups) including but not limited to the vestiges of racism, sexism, and classism.*^6^ Key questions teams should consider are (1) are the groups disproportionately impacted involved and to what capacity, what is their role and is there shared decision making and governance; (2) what is the equity capacity of the research teams and teams responsible for executing work of trials and dissemination; (3) have the researchers engaged in introductory and ongoing undoing racism/antioppression/racial equity opportunities and trainings; and (4) has the team participated in ongoing self-reflection of their privilege and power?

If community partners are involved in the process, the team should be expected to have an antiracist and equity-centered framework in which they approach their work; (5) is the institution involved in any practices or actions that have negatively affected Black and Brown communities or other communities disproportionately affected? For example, the University of Pittsburgh was involved in a historical review and name changing of the Graduate School of Public Health building formerly named after Thomas Parran, the first acting dean and former U.S. surgeon general during the Public Health Service Syphilis Studies (i.e., Tuskegee experiments).^7^

Some specific actions related to REMOVE include (1) examine the needs and priorities of the community, (2) create a clear timeline with proposed objectives and expectations and how they align with the needs and priorities of the communities, and (3) documentation of past engagement with Black and Latinx communities and efforts to provide support and mitigate existing inequities before and during COVID-19.

**REPAIR** considers the past by *taking into account the history that has led to current health inequities*.^6^ Some key questions teams should consider are (1) is there or will there be an account and accompanying statements acknowledging the historical trauma and oppression from the institutions involved in the vaccine trial studies institutions and their specific role that has led to current health inequities, particularly as it relates to Black and Latinx communities. (2) How will the institution specifically repair and intentionally invest in communities that were negatively affected by the institution? What is the role of the research and clinical team in this process? (3) What are some concrete ways that the research team will ensure those same actions will not be repeated?

Some actions related to REPAIR include an institutional and research team committee responsible for reviewing historical and contemporary actions that had a negative impact on Black and Brown communities and publicizing this information. In addition to institution and location-specific historical and contemporary contexts, the overall team should understand the larger body of work related to medical experimentation of Black and Brown communities including *Medical Apartheid: The Dark History of Medical Experimentation on Black Americans from Colonial Times to the Present* by Harriet Washington.^8^

**REMEDIATION** focuses on *addressing exposures happening currently and then buffering and protecting Black and Latinx from inequitable conditions*.^6^

Some key questions teams should consider are (1) will Black and Latinx people be compensated for their expertise in developing processes for research and dissemination of information and interventions (e.g., vaccinations); (2) will Black and Latinx participants have their medical expenses covered if they suffer a negative reaction from the vaccine during the trial or/and actual vaccine; and (3) currently, Black and Latinx people are disproportionately contracting COVID-19 and suffering from worst health outcomes with long-term implications. Will the institutions represented provide financial support *and* resources to mitigate the harm Black and Latinx communities are experiencing now due to COVID-19?

Specific actions to consider include working with local health systems, managed care organizations and insurance plans to provide the vaccine in ways that reduces burden with respect to costs and access (e.g., location and time). In addition, institutions should invest in community-led strategies and solutions that address the structural and social changes necessary to address not only COVID-19 inequities but also a multitude of other inequities.

**RESTRUCTURING** is addressing *structural and organizational inequities that systematically exclude certain groups and provide advantage to others*.^6^

Key questions teams should consider are (1) are specific groups excluded and eliminated from the planning, development, and the overall process; (2) what is the known power structure in terms of decision making and how can those power structures be interrogated and changed; (3) are those with power and privilege (typically the researcher and their institutions) willing to relinquish that power to achieve equity; and (4) specific to meetings and communication across groups, is the meeting structure excluding or privileging some over others, and can there be some creativity and flexibility in the time and frequency? A specific action item that teams should consider includes a coalition or collaborative to operate as an accountability collective, the overall process should begin with concerns and recommendations of community members, centering their expertise and experiences.

The final construct in the R4P framework is **PROVIDE**, or *centering service provision on culturally and economically sensitive strategies that address disadvantage of marginalized communities*.^6^ Some critical questions include (1) will there be critical services such as transportation to increase access for Black and Latinx communities to and from the clinic sites and (2) will vaccine clinics be opened on the evenings and weekends? Additional questions to consider are (3) how invested are the researchers in authentic community trust building and what is the capacity to do this within specific timelines such as trials and research studies; (3) will the coalition continue to conduct the work after the COVID-19 trials and vaccination distribution has been completed or resolved; and (4) will there be equitable compensation provided to community members?

Specific actions include the following: The vaccine team should provide ongoing and regular demographic data of the trial enrollment and vaccine distribution to the larger community along with other regular communication updates for transparency and accountability.

The questions and recommendations posed are not an all-inclusive list, as modifications should be made to address the local context. This tool can be used as a guiding framework for vaccine trial research, vaccine distribution, and other health-related concerns that extend beyond COVID-19. R4P is useful for developing a process to repair historical exploitation in medicine/health, remove and restructure practices and processes that exacerbate inequities and systems of oppression, and provide next steps for addressing health inequities.

## Ethical Approval

This article does not contain any studies with human or animal participants performed by any of the authors.

## Abbreviation Used

R4P: remove, repair, remediate, restructure, and provide
